# Grade 3 Dermatitis Secondary to Two Aromatase Inhibitors in Early Hormone Receptor-Positive Breast Cancer: A Case Report

**DOI:** 10.7759/cureus.97822

**Published:** 2025-11-26

**Authors:** Katherine Gayford, Nicola O'Neill, Ahmed Hadeya, Diego Ottaviani

**Affiliations:** 1 Oncology, University College Hospital, London, GBR

**Keywords:** anastrozole, aromatase inhibitor therapy, breast oncology, drug induced skin reactions, exemestane, hr+/erbb2− breast cancer, letrozole, non-steroidal aromatase inhibitor

## Abstract

Aromatase inhibitors (AIs) are widely used in the treatment of hormone receptor-positive breast cancer (HR+BC). AIs reduce the risk of HR+BC recurrence and improve disease-free survival (DFS) compared with other endocrine therapies (ETs). We report a rare case of an early, severe dermatological adverse event (dAE) to two AIs in a pre-menopausal patient receiving adjuvant treatment for human epidermal growth factor receptor 2 negative (HER2-) HR+BC. The patient developed a grade 3 eruptive, maculopapular dermatitis to exemestane and subsequently letrozole. The rash was poorly responsive to conservative management and only resolved following withdrawal of the AI. Unusually, a third AI - anastrozole - has been well tolerated. Severe dAEs secondary to AIs are rare, and there is a lack of evidence-based research to guide management of these reactions.

## Introduction

The suppression of oestrogen activity is an important strategy in the management of hormone receptor-positive breast cancer (HR+BC), applied in both adjuvant and metastatic settings [1-3]. Aromatase inhibitors (AIs) act by competitively inhibiting aromatase, an enzyme that converts androgens into oestrogen in peripheral tissues [1]. Third-generation AIs include anastrozole, letrozole, and exemestane. The non-steroidal AIs, anastrozole and letrozole, are reversible inhibitors of aromatase, whereas exemestane, a steroidal AI, is an irreversible inhibitor [1]. AIs do not prevent ovarian oestrogen production and are consequently given alongside ovarian suppression (OS) in pre-menopausal patients [2].

AIs are generally well tolerated but are associated with a range of adverse events (AEs), including hot flushes, vaginal dryness, reduction in bone mineral density and arthralgia. Dyslipidaemia, thrombo-embolic events and cardiovascular disease have also been reported [4].

AIs confer a significant advantage over tamoxifen (the previous standard of care) in the treatment of HR+BC in post-menopausal women [4-7]. The ATAC trial demonstrated that anastrozole improved disease-free survival (DFS) and reduced the risk of recurrence in post-menopausal women with HR+BC compared with tamoxifen [5]. The TEXT and SOFT trials have subsequently demonstrated that compared with tamoxifen, exemestane given alongside OS improved DFS and reduced risk of recurrence in pre-menopausal patients with human epidermal growth factor receptor 2 negative (HER2-) HR+BC [2,7]. In patients where exemestane is not tolerated, anastrozole and letrozole may be used as alternatives [3,4].

AI-induced dermatological adverse events (dAEs) are rare, with most evidence to date available from case reports or small case series. The reported dAEs include cutaneous systemic lupus erythematosus (SLE), cutaneous vasculitis, erythema nodosum and generalised rash [8-13]. A recent pharmacovigilance study has evaluated the incidence and type of AI-induced dAEs reported to the Food and Drug Administration Adverse Event Reporting System (FAERS) [14]. The most frequently reported dAEs were nail disorders and alopecia, but cutaneous reactions, including generalised dermatitis, cutaneous vasculitis, erythema nodosum and cutaneous SLE, were reported [14]. There is no consensus for the management of AI-induced dAEs, and the overall frequency is unknown. General management strategies for more severe dAEs have included withdrawal of the AI and the use of topical and/or systemic steroids [8-13].

Here, we report an unusual case of grade 3 eruptive dermatitis to both exemestane and letrozole, with subsequent sustained tolerance to anastrozole. This case highlights the need for improved awareness around dAEs secondary to AIs and supports consideration of trialling different drugs in this class for patients affected by dAEs.

## Case presentation

A 38-year-old female was diagnosed with a grade 3 (high-grade) invasive ductal carcinoma (IDC) in July 2024. Biopsy results demonstrated an HR+HER2-malignancy with a Ki67 score of 40%. She had undergone one unsuccessful cycle of in vitro fertilisation (IVF) in 2022, followed by a successful pregnancy in 2023. She had no other significant past medical history except for an allergy to gadolinium-based contrast agents and medical adhesives. She was pre-menopausal.

The patient underwent a right-sided mastectomy in September 2024, where two separate IDCs were removed. There were no distant sites of metastatic disease or lymph node involvement. She was staged as pT2(m)pN0M0. The patient's age, raised Ki-67, and high-grade disease put her at higher risk of recurrence [15-17]. Additionally, her Oncotype DX Breast Cancer Recurrence Score was high, at 26 [18]. On this basis, she completed adjuvant chemotherapy with four cycles of dose-dense epirubicin and cyclophosphamide followed by four cycles of paclitaxel. She did not undergo adjuvant radiotherapy.

**Figure 1:**
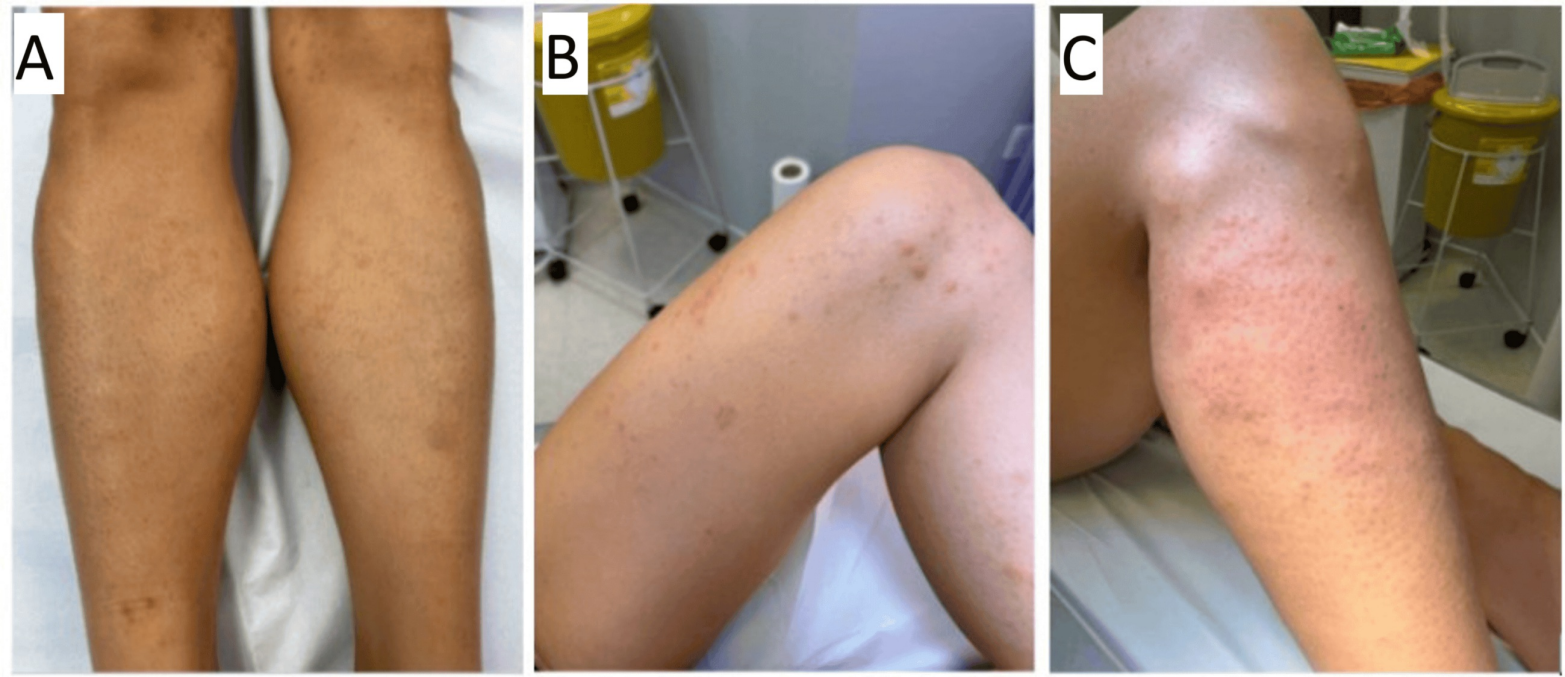
(A-C) The patient developed a generalised maculo-papular dermatitis one week after commencing ET with exemestane with goserelin. The rash was primarily distributed over the lower limbs. ET: endocrine therapy

In April 2025, the patient commenced endocrine therapy (ET) with exemestane 25mg once daily (OD) and 3.6mg goserelin once monthly as OS. Eight days after commencing exemestane, she developed an intensely pruritic, maculopapular rash distributed primarily over the lower limbs, with some truncal and upper limb involvement (Figure 1). Areas of confluence were noted over the lower limbs. The patient was referred to dermatology, who commenced topical dermovate and performed a punch biopsy on the left forearm. She continued exemestane.

**Figure 2:**
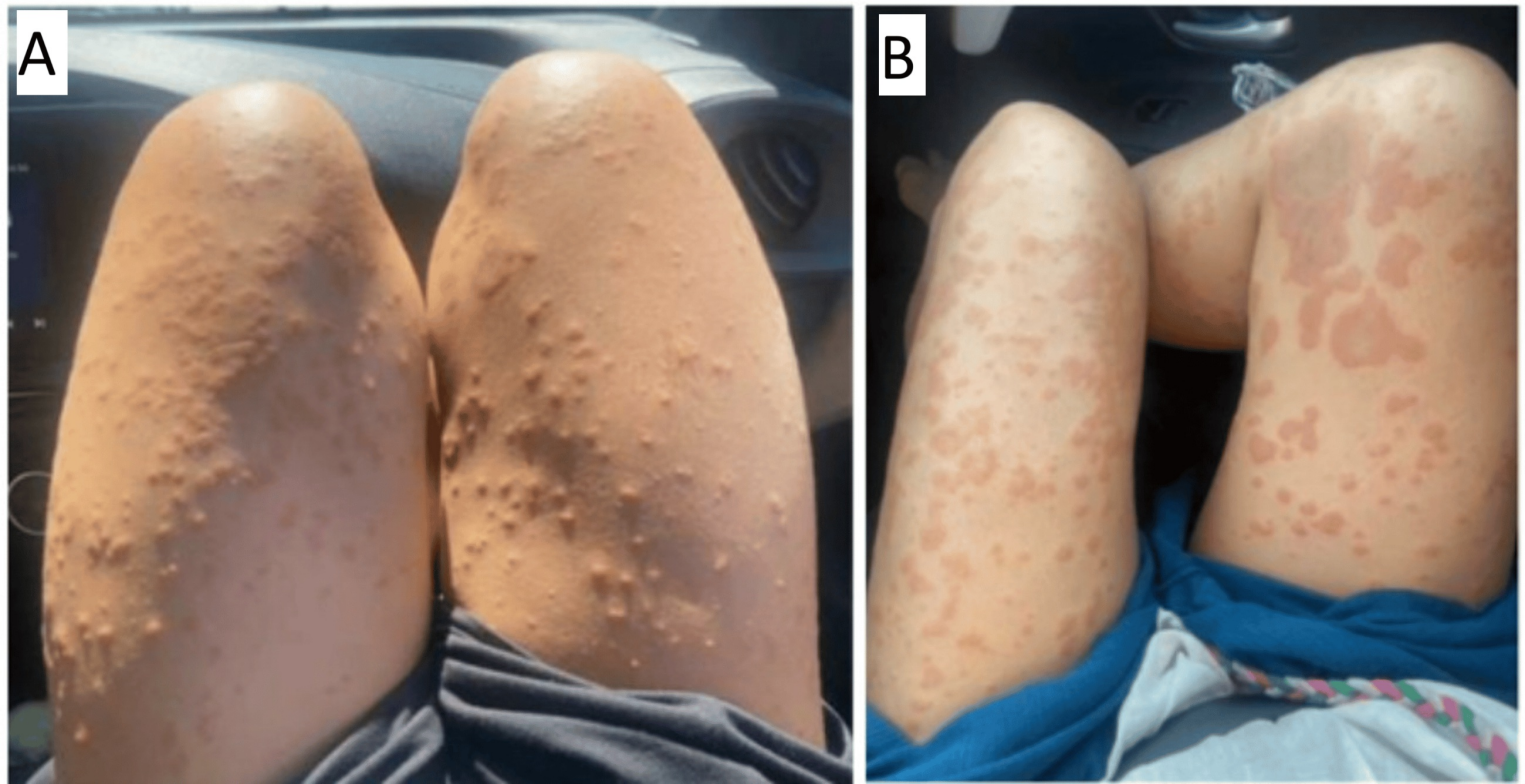
Two weeks after commencing endocrine therapy with exemestane and goserelin, there was a significant deterioration in the dermatitis despite treatment with topical corticosteroids. (A) Areas of urticaria distributed bilaterally over the anterior surfaces of the thighs. (B) Photograph taken two days after image A, demonstrating larger areas of urticaria involving the left thigh, right thigh, and anteromedial calf.

One week later, despite treatment with topical steroids and oral antihistamines, the rash had deteriorated significantly (Figure 2). The areas of skin involvement had become more confluent with more than 30% body surface area (BSA) involvement and areas of urticaria consistent with a grade 3 skin rash toxicity [19]. Systemic steroids were considered.

**Figure 3:**
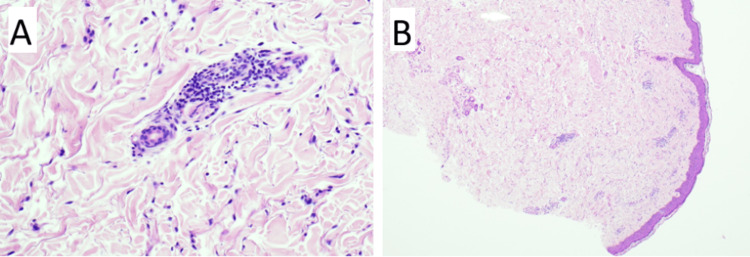
Histopathological slides from a biopsy taken from the left forearm one week after initiation of endocrine therapy with exemestane and goserelin. The biopsy demonstrated a mid-dermal, tightly cuffed perivascular lymphohistiocytic infiltrate with no epidermal involvement. The differential diagnosis included a dermal hypersensitivity reaction. (A) High-power view showing a perivascular lymphocytic infiltrate within the superficial dermis. (B) Low-power view demonstrating an intact epidermis overlying the dermis.

The biopsy taken one week previously demonstrated a mid-dermal tightly cuffed perivascular lymphohistiocytic infiltrate in the dermis, with no epidermal involvement (Figure 3). The biopsy findings were nonspecific, with differentials including a viral exanthem, erythema annulare centrifugum and a drug reaction. Blood test results, including C-reactive protein, full blood count, liver and renal function, were all unremarkable. There was no clinical or biochemical evidence of infection, and the dermatitis phenotype was not in keeping with annular centrifugum. Exemestane and goserelin were the only recently introduced drugs. Given the temporal correlation between dermatitis onset and initiation of exemestane, exemestane was stopped for a trial period. The patient experienced an immediate improvement in her symptoms on the withdrawal of exemestane. She continued topical dermovate.

**Figure 4:**
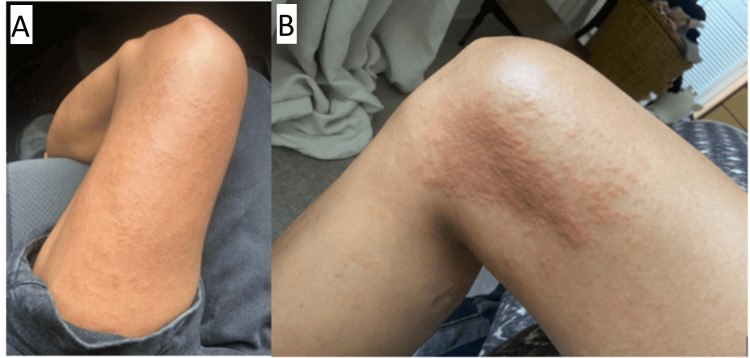
One week after the initiation of letrozole, there was a widespread recurrence of the dermatitis, including involvement of the lower limbs (as imaged). (A) Maculopapular dermatitis distributed over the anterior surface of the left thigh. (B) A discrete patch of dermatitis localised to the medial surface of the right knee.

Letrozole 2.5mg OD was selected as an alternative AI. One week after the initiation of letrozole, the patient experienced a recurrence of the rash, although the morphology had altered slightly (Figure 4). The rash was largely distributed over the lower limbs, but there was further involvement over the patient’s back and neck, with over 30% BSA involvement. Letrozole was stopped, and topical steroids were re-initiated. The patient experienced rapid improvement in her symptoms following cessation of letrozole.

Following the patient’s reaction to letrozole, she consulted with a Chinese Herbal Medicine practitioner. She was also receiving treatment with acupuncture. A third AI, anastrozole 1mg OD, was started in June 2025 without any rash recurrence. The patient was able to reduce her use of topical steroids and continues anastrozole without any major toxicities.

## Discussion

Our patient was at higher risk of recurrence based on her age, Ki-67 score and the histopathological grade of her tumours. The TEXT and SOFT trials demonstrated that in higher-risk HR+BC patients, AIs improve DFS and reduce the risk of recurrence [2,7]. Consequently, establishing treatment with an AI was a clinical priority for our patient. However, tamoxifen would have been considered as an alternative had no AI been tolerated.

dAEs represent a small proportion of AEs secondary to AIs [14]. There is no consensus for the management of AI-induced generalised maculopapular rash. While exemestane, a steroidal AI, and subsequently letrozole, a non-steroidal, resulted in the development of similar skin rashes, anastrozole continues to be well tolerated, and the underlying mechanism for this remains unclear. It is unusual to observe a cross-sensitivity to letrozole and exemestane, with subsequent tolerance to anastrozole. We identified only one similar case report in the literature describing a letrozole induced skin eruption that recurred upon letrozole re-challenge but did not recur with subsequent anastrozole therapy [20]. The pharmacological profiles of third-generation AIs differ, and therefore, we would suggest that physicians consider trialling alternative AIs in patients presenting with dAEs [21]. This approach could be considered even in relatively severe dAEs such as the one observed.

The recent FAERs database analysis provides the first large-scale study of dAEs secondary to AIs, providing new insight into the incidence, type and onset of dAEs seen in post-menopausal patients. A total of 2237 dAEs secondary to AIs were identified over 20 years, although search terms included nail disorders and hair growth changes, which were more frequently reported than cutaneous AEs [14]. Data obtained from the FAERS database suggests that letrozole is less frequently associated with dAEs. This may be due to its unique metabolism and comparatively longer half-life [14]. This was not observed in our patient. Prior to this study, research into dAEs secondary to AIs had been limited to a small number of case reports and case series [8-11]. Case reports have frequently focused on unusual dAEs, including SLE and cutaneous vasculitis, while histologically non-specific widespread eruptive rashes such as the one described have been less frequently reported [9-12].

Skin biopsy findings in our patient were non-specific, and the underlying pathogenesis of the rash was unclear, although a drug reaction was the leading histopathology differential. The temporal correlation between initiating AI treatment followed by the rapid development of a skin rash was important in identifying the aetiology of the patient’s rash. The Naranjo Adverse Drug Reaction (ADR) Scale for our patient was 5, suggesting a probable AD [22]. There is a lack of awareness regarding dAEs as a potential side effect of AIs, and consequently, they may be underreported due to diagnostic uncertainty.

## Conclusions

AIs have demonstrated a significant survival benefit for HR+BC and are generally well tolerated. Here, we have described a rare dAE from a steroidal and subsequently nonsteroidal AI. Anastrozole, a nonsteroidal AI, continues to be well tolerated. We would suggest clinicians consider trialling alternative AIs in patients presenting with dAEs, particularly in higher-risk patients who would benefit from treatment with an AI. The third-generation AIs have distinct pharmacological properties, and therefore, cross-sensitivity may not be universal as observed in our patient. While dAEs secondary to AIs are unusual, the number of cases identified in the FAERs database suggests that there may be a lack of discussion around AI-induced dAEs and their management. Reporting of such cases remains an important tool in raising awareness around these unusual reactions.
